# Prenatal Diagnosis of 4p and 4q Subtelomeric Microdeletion in De Novo Ring Chromosome 4

**DOI:** 10.1155/2013/248050

**Published:** 2013-12-19

**Authors:** Halit Akbas, Naci Cine, Mahmut Erdemoglu, Ahmet Engin Atay, Selda Simsek, Aysegul Turkyilmaz, Mehmet Fidanboy

**Affiliations:** ^1^Department of Medical Biology, Faculty of Medicine, Harran University, 63100 Sanliurfa, Turkey; ^2^Department of Medical Biology and Genetics, Faculty of Medicine, Dicle University, 21280 Diyarbakir, Turkey; ^3^Department of Medical Genetics, Faculty of Medicine, Kocaeli University, 41000 Kocaeli, Turkey; ^4^Department of Gynecology and Obstetrics, Faculty of Medicine, Dicle University, 21280 Diyarbakir, Turkey; ^5^Department of Internal Medicine, Bagcılar Education and Research Hospital, 34200 Istanbul, Turkey

## Abstract

Ring chromosomes are unusual abnormalities that are observed in prenatal diagnosis. A 23-year-old patient (gravida 1, para 0) referred for amniocentesis due to abnormal maternal serum screening result in the 16th week of second pregnancy. Cytogenetic analysis of cultured amniyotic fluid cells revealed out ring chromosome 4. Both maternal and paternal karyotypes were normal. Terminal deletion was observed in both 4p and 4q arms of ring chromosome 4 by fluorescence in situ hybridization (FISH). However deletion was not observed in the WHS critical region of both normal and ring chromosome 4 by an additional FISH study. These results were confirmed by means of array-CGH showing terminal deletions on 4p16.3 (130 kb) and 4q35.2 (2.449 Mb). In the 21th week of pregnancy, no gross anomalia, except two weeks symmetric growth retardation, was present in the fetal ultrasonographic examination. According to our review of literature, this is the first prenatal case with 4p and 4q subtelomeric deletion of ring chromosome 4 without the involvement of WHS critical region. Our report describes the prenatal case with a ring chromosome 4 abnormality completely characterized by array-CGH which provided complementary data for genetic counseling of prenatal diagnosis.

## 1. Introduction

Autosomal ring chromosomes are uncommon cytogenetic aberrations in prenatal diagnosis. Estimated frequency ranges from 1/27000 to 1/62000 in consecutive newborn and prenatal diagnosis studies [1]. The classic mechanism of ring formation is breakage in both arms of a chromosome followed by fusion of the two broken arms and loss of the distal segments. Therefore, phenotypic abnormalities associated with partial deletions can be found among patients with ring chromosomes [2–4]. Cases with ring chromosome 4 share common clinical features, including severe mental and motor retardation, cleft lip and palate, low birth weight, and microcephaly [5]. Diagnosis of ring chromosomes and identifying deleted segment, which is rarely diagnosed in the prenatal period, is of critical information for genetic counselling.

In the present study, our aim was to determine whether ring chromosome leads to abnormality in the following weeks of pregnancy or in the postnatal period of fetus which has no gross anomalia in the second trimester of pregnancy, except growth retardation, and the second aim was to manage genetic counselling to family members. For this purpose, we performed classical cytogenetic, molecular cytogenetic (FISH), and array-CGH techniques in amniocentesis and chordosynthesis samples to establish whether any deletion exists in ring chromosome 4 with breakpoints of deleted segments and identify genes involving deletions.

## 2. Case Report

**Figure 1:**
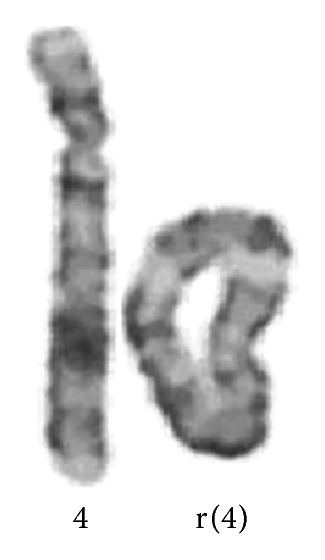
Partial G-banded karyotype shows normal chromosome 4 and ring chromosome 4.

A 23-years-old patient with a history of a pregnancy resulted with abortus referred for amniocentesis due to abnormal maternal serum screening test in the 16th week of the 2nd pregnancy. Down syndrome risk was 1,97 according to maternal serum screening test in the 12th week of pregnancy. Cytogenetic analysis of the cultured amniocytes using flask culture method revealed ring chromosome 4 [46,XX, r(4) [30 cells]] (Figure 1). To confirm the diagnosis, cytogenetic analysis of cord material obtained by cordocentesis was performed. Ring chromosome 4 was evident. Because parental caryotypes were normal, caryotype abnormality of case was considered de novo.

**Figure 2:**
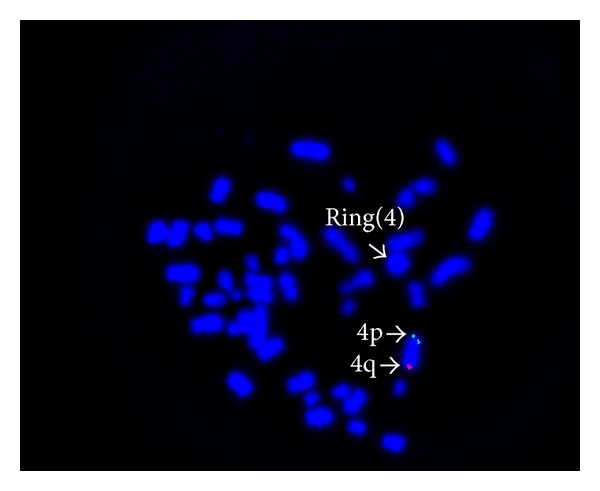
FISH study using a chromosome 4p specific subtelomeric probe (green signal) and a chromosome 4q specific subtelomeric probe (red signal) shows that subtelomeric 4p and 4q are deleted in ring chromosome 4.

**Figure 3:**
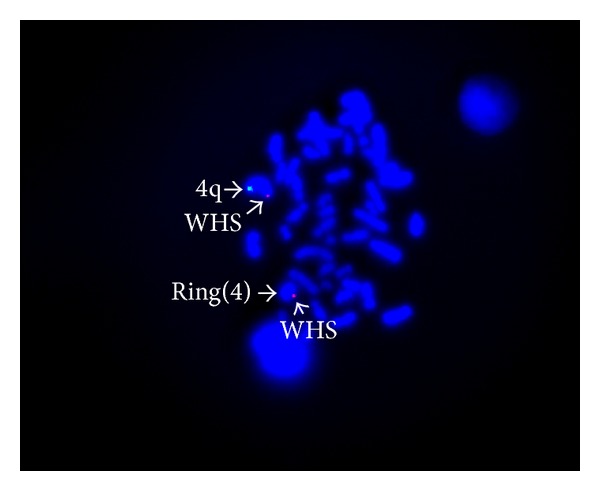
FISH study using a WHS critical region probe (red signal) and chromosome 4q subtelomere specific control probe (green signal) shows the presence of WHS critical region on both normal and ring chromosomes 4.

The ring chromosome 4 was characterized by FISH using the 4p and 4q specific subtelomeric probe (Chromoprobe Multiprobe-T System; Cytocell Ltd, UK). FISH study showed deletions at the subtelomeric regions of 4p and 4q on the ring chromosome 4 (Figure 2). WHS critical region probe with 4q subtelomere specific control probe was used to establish the diagnosis of deletion in the Wolf Hirschhorn critical region in the second FISH study (WHS Critical Region Probe, Cytocell). However deletion was not observed in WHS critical region of ring chromosome 4 (Figure 3).

Array-CGH was performed to determine breaking points and involved genes on terminal deletions of ring chromosome 4.

**Figure 4:**
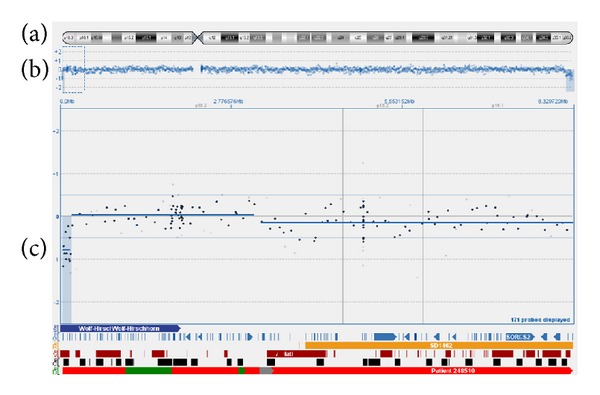
Array-CGH result of chromosome 4: 4p16.3→pter deletion. There are three icons from top to down in total (a)–(c). (a) A diagram of chromosome 4. (b) A scatter plot of a copy number; each point represents the mean copy number calculated from consecutive 100 probe sets. The baseline in the middle indicates the normal copy number level. Upward deviation from the baseline indicates amplification and downward departure from the baseline represents deletion. (c) Enlarged form of 4p16.3→pter segment at (b) region; combed region represents copy number variants (CNV) that matches deleted segment.

**Figure 5:**
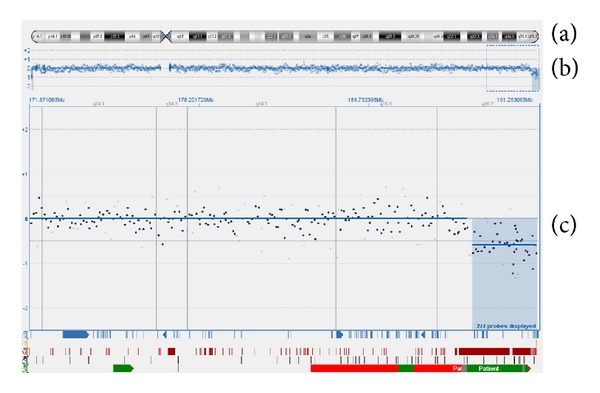
Array-CGH result of chromosome 4: 4q35.2→qter deletion. There are three icons from top to down in total (a)–(c). (a) A diagram of chromosome 4. (b) A scatter plot of a copy number; each point represents the mean copy number calculated from consecutive 100 probe sets. The baseline in the middle indicates the normal copy number level. Upward deviation from the baseline indicates amplification and downward departure from the baseline represents deletion. (c) Enlarged form of 4q35.2→qter segment at (b) region; combed region represents copy number variants (CNV) that matches deleted segment.

CytoSure microarray platform (Oxford Gene Technology) was used for aCGH analysis. This platform has approximately 44,000 oligonucleotide probes. The arrays were scanned on an Agilent G2505B scanner and quantified using Agilent's Feature Extraction software. Data was then normalised using CytoSure visualisation software which uses a standard LOWESS method [6]. Subsequent to array-CGH, a deletion of 130 Kb involving 6 genes in 4p16.3 [arr 4p16.3(Start: 34021 - Stop: 164174x1)] and a second deletion of 2.449 Mb involving 17 genes in 4q35.2 [arr 4q35.2(Start: 188713284 - Stop: 191162284x1)] were determined (Figures 4 and 5). The list of deleted genes was shown in Table 1. Deletion range in 4p determined by array-CGH (Start: 34021 - Stop: 164174) was out of range of FISH probe which was used for WHS critical region (Start: 1.894.975 - Stop: 2.117.567). Eventually, the results of FISH and array-CGH confirm each other.

In the 21th week of pregnancy, no gross anomalia, except 2 weeks symmetric growth retardation, existed in the fetal ultrasonographic examination. Parents were informed about the probable prenatal and postnatal complications of subtelomeric deletion of p and q arms of chromosome 4. However, parents denied to terminate pregnancy.

## 3. Discussion

In the present report, prenatal case with 4p16.3 and 4q35.2 subtelomeric deletion of ring chromosome 4 without the involvement of WHS critical region was diagnosed.

Ring chromosome 4 is an uncommon cytogenetic abnormality that has been rarely diagnosed in the prenatal stage. Up to now, three cases with prenatal diagnosis of ring chromosome 4 were reported in the English literature. In the first case, Sherer et al. reported a severely and symmetrically growth-retarded fetus with microcephaly, hypertelorism, and hypoplastic genitalia with a two-vessel umbilical cord, a characteristic “Greek warrior helmet” facial profile and clubfoot in a 29th week fetus of ring chromosome 4 carrier with 4p15 and 4q35 deletions [7]. In the second case, Chen et al. reported a case with intrauterine growth retardation, increased nuchal translucency, and a suspected cardiac malformation in a 17th week fetus of ring chromosome 4 carrier with 4p15.2 and 4q35.2 deletions [8]. The third case was a female fetus at the 21st week of gestation with ring chromosome 4 (p16;q33) which presented with further abnormalities, namely, cleft lip, left-sided diaphragmatic hernia with cardiac dextroposition, a single umbilical artery, and pathological uterine blood flow patterns [9]. In all of 3 cases, deletions involve larger segments than those in our case and deleted 4p segment involved WHS critical region that was responsible from the development of Wolf-Hirschorn Syndrome. In contrast, 4p16.3 deletion in our case involves a small segment of 130 kb and does not involve WHS critical region. Fetal ultrasonographic examination pointed out growth retardation of two weeks without an accompanying abnormality. This may be related to absence of deletion in the WHS critical region and shortness of deleted segments than previous three cases.

Among published cases with 4p16 deletions, South et al. reported a case of ring 4 with 1.27–1.46 Mb deletion at 4p16.3 presented with significant postnatal growth retardation, mild developmental retardation, and nutritional disturbances. In addition to abnormalities of the first case, the second reported that a case exhibited terminal 4p microdeletion of approximately 1.78 Mb caused complex seizure disorder. No deletion was observed at WHS region in both cases [10]. Khonsari et al. reported a case with deletion of 300 kb from telomere presented with multiple vascular malformations, unilateral syndactyly, and bilateral macrodactyly in the postnatal period [11]. Blackett et al. reported another case of ring chromosome 4 with a deletion of 145 kb at 4p16 that has mild growth retardation, deafness, short stature, obesity, and early onset of type 2 diabetes [12]. Furthermore, small 4p deletions located approximately 100–300 kb from 4p ter are reported without phenotypic modification [13]. Another case had subtelomeric 4q35.2 deletion that encompasses approximately 3 Mb with comorbid schizoaffective disorder and mental retardation [14]. It is possible that our patient may experience similar abnomalies in the later periods of pregnancy and postnatal period.

In the present case, genes that encode 6 zinc finger protein exist on deleted segment of 130 kb at 4p16.3 as shown in Table 1. To the best of our knowledge, no anomalia that is related to these genes existed in the literature. On the other hand, seventeen genes exist on deleted segment of 2.449 Mb at 4q35.2. Among the genes that were shown in Table 1, only FRG1 and FRG2 genes were considered to relate with facioscapulohumeral muscular dystrophy [15]. Hemizygosity of deleted 23 genes on 4p and 4q of ring chromosome have risk of clinical pathologies at the postnatal period. Parents were informed about the risk of anomalies.

According to our knowledge, this is the first de novo ring chromosome 4 case with 4p and 4q subtelomeric deletion without deletion of WHS critical region and that had no fetal anomalia except intrauterine growth retardation determined by second trimester fetal ultrasonographic examination. In the light of literature, genetic counselling was performed to family members by considering genes on deleted segments. The role of FISH and array-CGH on genetic diagnosis and phenotypic correlation in the prenatal evaluation is confirmed.

## Figures and Tables

**Table 1:** Deletions and related genes of ring chromosome by Array-CGH.

Cytogenetic location of deleted segments	Genes	Bioinformatic data of deleted genes according to genome database*
4p16.3→pter	ZNF595	Zinc finger protein 595 may be involved in transcriptional regulation. Function: DNA binding, metal ion binding.
	ZNF718	Zinc finger protein 718 may be involved in transcriptional regulation, regulation of transcription.
	Z95704.1	Zinc finger protein 718 may be involved in transcriptional regulation.
	AC118278.4-2	Zinc finger protein 718 may be involved in transcriptional regulation, regulation of transcription.
	AC118278.4-1	Zinc finger protein 718 may be involved in transcriptional regulation, regulation of transcription.
	AC108475.5	Zinc finger protein 876: pseudogene, function: DNA binding, metal ion binding.

4q35.2→qter	AC093909.2:	Long intergenic nonprotein coding RNA 1060
	AC097521.2-1:	uncharacterized
	AC097521.2-2:	uncharacterized
	AC115540.3	ADAM metallopeptidase domain 20 pseudogene 3.
	AC108073.3-1	ZFP42 zinc finger protein. Function: DNA binding, metal ion binding.
	AC108073.3-2,	TRIML2: tripartite motif family-like 2. Function: ligase activity.
	AC108073.3-3:	FAUP3: FBR-MuSV-associated ubiquitously expressed (fox derived) pseudogene 3.
	ZFP42	Zinc finger protein. Function: DNA binding, metal ion binding.
	TRIML2	Tripartite motif family-like 2. Function: igase activity.
	U6:	RNA; U6 small nuclear 1
	TRIML1,	Tripartite motif family-like 1. Function: ligase activity, zinc ion binding.
	HSP90AA4P,	Heat shock protein 90 kDa alpha (cytosolic), class A member 4, pseudogene. Function: unfolded protein binding, ATP binding.
	U1,	small nuclear RNA.
	AF250324.1-1,	uncharacterized
	FRG1	FSHD region gene 1. This gene is deleted in facioscapulohumeral muscular dystrophy (FSHD).
	TUBB4Q,	Tubulin, beta 7, pseudogene.
	AF146191.1-2:	FRG2: FSHD region gene 2. This gene is related with facioscapulohumeral muscular dystrophy (FSHD).

*http://www.ncbi.nlm.nih.gov/gene/.
